# *Ex Vivo* Expansion of Human Mesenchymal Stem Cells in Defined Serum-Free Media

**DOI:** 10.1155/2012/123030

**Published:** 2012-05-07

**Authors:** Sunghoon Jung, Krishna M. Panchalingam, Lawrence Rosenberg, Leo A. Behie

**Affiliations:** ^1^Pharmaceutical Production Research Facility (PPRF), Schulich School of Engineering, University of Calgary, Calgary, AB, Canada T2N 1N4; ^2^Department of Surgery, McGill University, Montreal, QC, Canada H3G 1A4

## Abstract

Human mesenchymal stem cells (hMSCs) are presently being evaluated for their therapeutic potential in clinical studies to treat various diseases, disorders, and injuries. To date, early-phase studies have indicated that the use of both autologous and allogeneic hMSCs appear to be safe; however, efficacy has not been demonstrated in recent late-stage clinical trials. Optimized cell bioprocessing protocols may enhance the efficacy as well as safety of hMSC therapeutics. Classical media used for generating hMSCs are typically supplemented with ill-defined supplements such as fetal bovine serum (FBS) or human-sourced alternatives. Ideally, culture media are desired to have well-defined serum-free formulations that support the efficient production of hMSCs while maintaining their therapeutic and differentiation capacity. Towards this objective, we review here current cell culture media for hMSCs and discuss medium development strategies.

## 1. Introduction

Human mesenchymal stem cells (hMSCs), also referred to as mesenchymal stromal cells [1], demonstrate regenerative properties and multipotentiality, and thus have been proposed as a potential candidate for cell therapies and tissue engineering. Clinical studies employing hMSCs derived from different sources have been initiated for the treatment of several diseases and injuries such as myocardial infarction, osteogenesis imperfecta, graft-versus-host disease (GVHD), and Crohn's disease, spinal cord injury, multiple sclerosis, and diabetes (http://www.clinicaltrials.gov/). Early-phase studies with thousands of patients have indicated that the use of both autologous and allogeneic hMSCs appears to be safe; however, efficacy has not been demonstrated in recent late-stage clinical trials [2]. In general, clinical protocols employ cell culture technologies by which a small fraction of primary hMSCs are isolated from a selected tissue source and expanded for multiple passages in order to generate a clinically relevant number of cells. Consequently, once the tissue source of hMSCs is determined for an intended clinical application, the safety and efficacy of cell therapeutics produced may be significantly influenced by cell bioprocessing protocols [3]. As a consequence, developing robust production processes by optimizing culture variables is critical to efficiently and consistently generate hMSCs that retain desired regenerative and differentiation properties while minimizing any potential risks.

Cell culture variables include medium formulation (basal media and supplements), culture surface substrate, cell seeding density, physiochemical environment (dissolved oxygen and carbon dioxide concentrations, temperature, pH, osmolality, and buffer system), along with subculture protocols. In particular, the development of well-formulated culture media for both the isolation and expansion of hMSCs is imperative, but has been recognized as an extremely difficult process due to the high complexity of media formulations. Herein, we review various types of media that are currently used for clinical studies or under evaluation, along with the biological characteristics and *ex vivo *expansion procedures for hMSCs. It is clear that defined media optimized for hMSC isolation and expansion would greatly facilitate the development of robust, clinically acceptable bioprocesses for reproducibly generating quality-assured cells. Although several serum-free formulations have recently been developed, the performance of most media seems to be suboptimal. Identifying critical factors and their concentrations towards designing an ideally formulated, chemically defined serum-free medium should be carried out using rational and systematic approaches. Hence, in the second part of this paper, we discuss crucial strategies and important decisions needed for serum-free medium development.

## 2. Mesenchymal Stem Cells

### 2.1. What Is an MSC?

Friedenstein and colleagues first reported a small fraction of cells in bone marrow (BM) attached to and proliferated on tissue culture substrates and that these cells were able to differentiate into multiple cell types such as adipocytes, osteoblasts, and chondrocytes both *in vitro* and *in vivo *[reviewed in [4]]. These cells were fibroblastic spindle-shaped and readily generated single-cell-derived colonies and were originally referred to as colony-forming unit-fibroblasts (CFU-F). Later, these cells were demonstrated by many investigators to be heterogeneous populations of nonhematopoietic adult stem/progenitor cell-like cells residing in marrow stroma and, thus, were called marrow stromal cells, mesenchymal stem cells, or multipotent mesenchymal stromal cells [1, 4]. In addition to BM, similar MSC-like cells have also been shown to be present in most tissues, including adipose tissue (AT), synovial membranes, bone, skin, pancreas, blood, fetal liver, lung, and umbilical cord blood (UCB) [4, 5].

### 2.2. Characteristics of hMSCs

BM has been the traditional source of hMSCs for basic research and therapeutic use because BM harvest is a routine and safe procedure. Therefore, the characteristics of *ex vivo* expanded hMSCs described below mostly represent BM-derived hMSCs unless otherwise stated.

#### 2.2.1. Morphology

Typically, hMSCs isolated and expanded in classical FBS-containing media are mostly spindle-shaped (or fusiform) and cuboidal fibroblast-like cells. More specifically, Prockop and colleagues demonstrated that hMSCs undergo a time-dependent morphological transition from thin (small), spindle-shaped cells (considered stem cells or early progenitors) to wider (larger), spindle-shaped cells (looked like more mature cells) when cells are plated at 1 to 1,000 cells/cm^2^ [6]. They further showed that the small, spindle-shaped cells proliferate more rapidly and have a higher level of multipotentiality, compared to the slowly replicating large cells, which have lost most of their multipotentiality but can still differentiate into a lineage (e.g., osteogenic) as a default pathway. The morphology (and size) of hMSCs may also be dependent upon culture conditions (e.g., growth media, culture surface). For example, hMSCs expanded in bFGF-supplemented media were smaller and proliferated more rapidly, compared to those in bFGF-lacking control conditions [7]. Culture surfaces (e.g., treated with Matrigel) might also affect the morphology [8].

#### 2.2.2. Growth and Adherence Characteristics

hMSCs are anchorage-dependent cells, which attach to a plastic surface, spread out, and grow, when maintained in standard culture conditions (e.g., DMEM supplemented with 10% FBS). The initial growth of hMSCs in primary BM cell culture on a plastic surface is characterized by the formation of single-cell-derived colonies, when the cells are plated at appropriate numbers. The cells in colonies generated in the primary culture can typically be subcultured through multiple passages at various plating densities. In general, hMSCs have great propensity for expansion in culture, although their proliferation potential is highly variable, depending on many aspects such as donor age, tissue source, and culture conditions. For example, Sekiya et al. demonstrated that hMSCs proliferate more rapidly when passaged by plating the cells at low densities (e.g., 10–100 cells/cm^2^, compared to 1,000–10,000 cells/cm^2^) [6]. hMSC proliferation is also highly variable depending on growth media [9].

#### 2.2.3. Immunophenotype

Currently, no prospective markers exclusively defining hMSCs are available. In general, hMSCs are negative for hematopoietic surface markers including Cluster of Differentiation (CD) 34, CD45, CD14, CD11b, CD19, CD79*α*, CD31, CD133 and positive for CD63, CD105, CD166, CD54, CD55, CD13, CD44, CD73, and CD90 [10, 11]. However, differences exist among the studies reporting the surface marker characteristics, which may be explained by variations in culture methods and/or differentiation stage of the cells [11].

#### 2.2.4. Multilineage Differentiation Potential

hMSCs have at least trilineage differentiation potential *in vitro* (i.e., the ability to differentiate into bone, cartilage and fat upon proper induction conditions). This is the most well-established characteristic of hMSCs and thus is considered the hallmark of these cells [12]. It has also been observed that hMSCs could give rise to other mesodermal cells and nonmesodermal cell types, such as neuron-like and endoderm-like cells [11].

#### 2.2.5. Minimal Criteria for Defining hMSCs

Standard culture protocols for the isolation and expansion of hMSCs have not been well established, in particular for growth medium. Therefore, different laboratories often use various methods of isolation/expansion and also different approaches to characterize the cells. This makes it difficult to compare and contrast the outcomes from various investigators. To minimize the variations, the Mesenchymal and Tissue Stem Cell Committee of the International Society for Cellular Therapy has proposed minimal criteria to define hMSC populations. These include the following: (i) hMSCs must be plastic-adherent when maintained in classical culture conditions; (ii) hMSCs must express high levels (≥95% positive) of CD105, CD73, and CD90 and lack expression (≤2% positive) of CD45, CD34, CD14, or CD11b, CD79*α* or CD19, and HLA-DR (unless stimulated by interferon-*γ*) surface molecules; (iii) hMSCs must differentiate into osteoblasts, adipocytes, and chondroblasts under specific *in vitro* differentiation conditions [10].

### 2.3. Potential Therapeutic Properties of hMSCs

hMSCs show various properties that could be important in therapeutic applications. Indeed, some of these functions are currently being exploited in clinical trials with great promise.

#### 2.3.1. Multipotentiality

As described earlier, hMSCs can differentiate into distinctive mesenchymal phenotypes, and thus they have been used to reconstruct damaged tissues upon transplantation in association with scaffolds. In addition, due to their differentiation potential into nonmesodermal cell types, hMSCs cells have also been proposed for replacement therapies to treat various diseases and disorders such as neuronal diseases and diabetes [13, 14].

#### 2.3.2. Tropism for Sites of Disease

hMSCs appear to be capable of homing to sites of disease or damage. It has been reported that hMSCs systematically infused into diabetic mice migrated to damaged sites (i.e., pancreatic islets and renal glomeruli) and contributed to the repair of tissue [15] by certain mechanism(s) yet unidentified.

#### 2.3.3. Secretion of Bioactive Factors

hMSCs inherently synthesize and secrete a broad range of bioactive agents such as cytokines and growth factors [16]. This intrinsic secretory activity of hMSCs may significantly contribute to tissue repair or regeneration, presumably by establishing a regenerative microenvironment at sites of tissue injury or damage [16]. Originally, therapeutic effects observed with the use of hMSCs were thought to be due to their transdifferentiation (i.e., differentiation into nonmesodermal cell types) potential. However, these beneficial effects were often demonstrated without evidence for the engraftment and transdifferentiation of transplanted hMSCs in animal model studies. Therefore, these indirect, secretory functions of hMSCs have been proposed as an alternative mechanism explaining the therapeutic effects, and importantly, these characteristics have generated clinical interest to use undifferentiated hMSCs for various applications such as repair or regeneration of damaged tissues [16].

#### 2.3.4. Immunomodulation

hMSCs also display immune regulatory properties that might represent a critical role in the therapeutic application of these cells [17]. *In vitro* studies using hMSCs demonstrated that these cells suppress the proliferation of T cells. Further, it was also revealed that hMSCs inhibit the differentiation and maturation of dendritic cells (DCs) and decrease the production of inflammatory cytokines by various immune cell populations [18]. DCs are the most potent antigen-presenting cells, which specialize in antigen uptake, transport, and presentation and have the unique capacity to stimulate naïve and memory T cells [17]. In addition to the *in vitro* effects, it has been seen in animal model studies that hMSCs may also display immunosuppressive capacities *in vivo*. For example, hMSCs facilitated engraftment of hematopoietic stem cells and prolonged skin allograft survival [19]. Further, it has been demonstrated that the use of hMSCs reversed severe acute GVHD [19]. However, while *in vitro* results consistently show the immunosuppressive capacity of hMSCs, studies in animals and humans suggest that hMSCs are less effective in producing systemic immunosuppression *in vivo *[20]. Therefore, further studies using standardized hMSC populations are urgently needed to verify their *in vivo* immunosuppressive potential and to define the optimal conditions for the use of hMSCs as immunotherapy.

#### 2.3.5. Immune Privileged or Hypoimmunogenic Property

When undifferentiated hMSCs were transplanted into recipients in preclinical and clinical trials, these cells produced various cytokines and growth factors and had an ability to modify the response of immune cells. Another important observation is that hMSCs escape immune recognition or at least possess a hypoimmunogenic character upon allogeneic transplantation [21]. Indeed, clinical studies showed that hMSCs evoke little or no immune reactivity in allogeneic recipients [5]. This indicates that, in addition to the transplantation of autologous cells to patients to minimize the risk of immune response, allogeneic hMSCs could also be safely used. If this is the case, the use of allogeneic hMSCs has an important advantage in that the culture-expanded cells could be considered as an “off the shelf” therapeutic product. Indeed, clinical studies using allogeneic, as well as autologous, hMSCs from BM have been initiated for the treatment of several diseases and injuries such as osteogenesis imperfecta, GVHD, leukemia, myocardial infarcts, Crohn's Disease, cartilage and meniscus injury, stroke, and spinal cord injury [5, 16].

### 2.4. Safety of Using hMSCs

A large body of safety data has been gained from the use of hMSCs in various clinical applications including the treatment of GVHD and the facilitation of BM engraftment [14]. That is, until present, only few adverse effects attributed to hMSC transplantation have been reported [22]. This has facilitated the rapid translation of basic research into clinical trials. However, there are some obvious issues that need to be addressed before the wide implementation of clinical trials using hMSCs.

#### 2.4.1. Tumor Formation

In general, it is considered that hMSCs can be safely cultured *in vitro* with no risk of malignant spontaneous transformation [23]. Stenderup and colleagues cultured several strains of hMSCs from BM at various ages (i.e., aged 18–81 years) until the cells reached their maximal life span without any evidence of transformation [24]. Further, there have been no reports with human trials demonstrating the formation of tumors by culture-expanded hMSCs [22]. Nonetheless, a potential risk for spontaneous transformation associated with hMSC proliferation *in vitro*, in particular after long-term culture, cannot be ruled out. The transplantation of primitive stem/progenitor cells with considerable proliferative potential can raise the possibility of tumor formation, and any *ex vivo* manipulation will increase the chances of transformation [14]. It has been recommended by the Food and Drug Administration (FDA) that “minimally manipulated” cells be used for human clinical trials. In this regard, attempts are being made to develop an efficient production system to produce clinically relevant numbers of hMSCs at relative shorter periods of time with lower passage numbers [25].

#### 2.4.2. Promotion of Tumor Growth and Development

A potential risk of treatment with hMSCs can paradoxically arise from the fact that these cells are capable of suppressing various immune cells, which may promote tumor growth and metastasis. The role of hMSCs in tumors is controversial: some studies have demonstrated enhancement of tumor growth and metastasis, while others have shown no apparent effect or inhibition of tumor growth with the use of hMSCs [26].

#### 2.4.3. Immune Response

Although hMSCs themselves appear to escape immune recognition, those “cultured in medium containing FBS” can produce immune reactions in patients receiving repeated administrations of these cells [27]. Conventionally, the optimal conditions for hMSC expansion require media supplemented with FBS at a concentration of 10–20%, which corresponds to approximately 5–10 mg FBS proteins/mL of medium. Spees et al. demonstrated that, when hMSCs were cultured in medium containing 20% FBS and harvested, 7–30 mg of FBS proteins were still associated with a standard preparation of 100 million hMSCs, a dosage that probably will be needed for clinical therapies [28]. Thus, immunological reactions caused by medium-derived FBS proteins will be a concern, in particular for certain types of cell therapy involving multiple administrations of hMSCs. The safety issue associated with the use of FBS in culture media will be avoided by developing an alternative culture protocol to produce hMSCs *in vitro* in the absence of FBS.

### 2.5. Sources of hMSCs

Although the traditional source of hMSCs is BM, it has been demonstrated that cells displaying similar characteristics with BM-hMSCs can also be derived from other sources including AT, UCB, umbilical cord tissue, placenta, amniotic fluid, liver, lung, pancreas, and muscle [29–34]. The ideal source of hMSCs for therapeutic use would be one that is readily available and can be expanded in culture rapidly to yield large numbers of cells. In this regard, hMSCs from readily obtainable tissue otherwise discarded, such as AT, may offer a preferable alternative to BM, because the collection of BM is an invasive procedure. Moreover, AT is a source of abundant hMSCs, and the AT-derived hMSCs have shown various potential therapeutic properties both *in vitro* and *in vivo* [29]. UCB, umbilical cord tissue, and placenta also represent attractive sources of hMSCs because these tissues are readily available and the derived cells may contain less genetic abnormalities and greater proliferative capacity than adult tissue-derived hMSCs [30–32].

It is important to note that there is much evidence demonstrating that hMSCs (or similar populations) derived from various sources showed different characteristics in gene expression profile, proliferation and differentiation potential, and functional properties although most of these satisfied the minimal criteria for defining MSCs and, thus, were considered as hMSCs as a whole [35–38]. For example, studies have shown that AT-hMSCs exhibited *in vitro* immunomodulatory properties at higher efficiencies, compared to BM-derived counterparts [35]. Another example can be found from a study comparing the differentiation potential of hMSCs from BM and pancreas into insulin-producing endocrine cells [36]. This study revealed that hMSCs derived from the pancreas are committed to an endocrine fate and thus have a greater propensity to generate insulin-producing cells compared to BM-hMSCs. Therefore, to select an ideal source of hMSCs for therapeutic use, their functional properties (e.g., differentiation potential, immunomodulation, secretion of bioactive factors) should be critically evaluated in comparison with those from other potential sources, in addition to the availability of tissue and cell proliferation capacity as mentioned earlier.

## 3. Generation of Mesenchymal Stem Cells

### 3.1. hMSC Number

The frequency of hMSCs in BM is very low. The CFU-F assay is widely used to estimate the number of MSCs in primary BM cells as well as passaged cell populations in culture [39, 40]. Using this assay, it has been reported that MSCs represent 0.01% to 0.001% of human BM mononuclear cells (MNCs) [39]. It was also demonstrated that hMSCs comprise approximately 1 in 10,000, 100,000, and 250,000 BM-hMNCs of newborns, teens, and 30 year-olds, respectively, indicating that the hMSC frequency is highly variable with age [16].

There is a significant difference in the frequency of hMSCs present in other tissues/organs, heavily depending on the source. Kern et al. reported different frequencies of hMSCs in BM, AT, and UCB. In their study, with culture-initiating populations (i.e., MNCs of BM and UCB, and stromal vascular fraction of AT), it was demonstrated that the number of CFU-Fs (i.e., corresponding to hMSCs) calculated at the basis of 1 × 10^6^ initially plated cells was highest for AT (557), followed by BM (83) [37]. In contrast, the frequency of CFU-Fs in UCB was considerably lower. Similar observations were reported by others [41, 42].

Although the dosage of hMSCs for their optimal use in therapeutic applications is still unclear and should be dependent upon the type of cell therapy, at least 1 to 2 × 10^6^ hMSCs per kg body weight of the adult patient is generally suggested [43]. Consequently, the number of primary hMSCs, regardless of the sources, is insufficient for research as well as clinical use. Hence, it is necessary to isolate hMSCs and then subsequently expand them for multiple passages on tissue culture substrates in order to generate clinically relevant numbers of cells.

### 3.2. Isolation and Expansion of hMSCs

#### 3.2.1. Isolation of hMSCs (Primary Culture)

As there are no universal surface markers available for exclusively defining hMSCs, these cells have traditionally been isolated from initial primary cell fractions (e.g., BM MNCs), based on their selective adherence, compared to hematopoietic cells, to plastic surfaces. Therefore, the hMSCs obtained are intrinsically heterogeneous. Importantly, it has been demonstrated that the characteristics of isolated cells are highly dependent upon culture conditions used. For BM cells, either unfractionated whole BM, fractionated MNCs by density gradient, or separated cells by depletion of certain subpopulations are used, with the fractioned MNCs being the most popular. 

#### 3.2.2. Expansion of hMSCs

As a large number of hMSCs is needed for their clinical use, cells obtained from the primary culture are further expanded through multiple passages. Typically, hMSCs obtained from young donors can undergo 24–40 population doublings (PD) in culture before they reach senescence, while those from older donors retain reduced proliferative potential [11]. Similar to other diploid cells, hMSCs grow at a rather constant rate during early passages (typically for the first 2 to 3 weeks) and then with a gradual increase in cell doubling times as the passage number increases until the growth ceases due to senescence [11]. It is also known that, after the initial culture, hMSCs progressively show loss of multipotentiality [44] under classical media and possibly other culture conditions.

### 3.3. Culture Media for hMSCs

#### 3.3.1. Classical FBS-Based Media

Conventional media used for isolating and expanding hMSCs include supplementation of FBS at 10–20% (v/v). FBS contains a high content of attachment and growth factors as well as nutritional and physiochemical compounds required for cell maintenance and growth. The function and characteristic of serum is further reviewed later in this paper. FBS-based media remain a common standard in generating hMSCs for basic research and clinical studies; however, the use of FBS is not desirable, raising several safety and other concerns. The inherent potential problems associated with the ill-defined FBS and other animal-derived supplements are as follows [45–47]:

- risk of contamination associated with harmful pathogens such as viruses, mycoplasma, prions, or unidentified zoonotic agents and transmissions of these contaminants to cells being used for cellular therapy,
- high content of xenogeneic proteins that can be associated with cell therapeutics during culture, causing concerns relating to immune reaction in patients, as described earlier,
- high degree of batch-to-batch variation causing inconsistency in the generation of quality-assured cells and thus making standardization of the production process difficult,
- presence of growth inhibitors, cytotoxic substances, and/or differentiation agents. (Beyond its growth-promoting property, serum may also contain components that are inhibitory for the growth of certain cell types. It has been demonstrated that some cell types cannot be cultured in the presence of serum at its typical concentrations in medium due to its unidentified cytotoxic constituents. It is also well known that serum is toxic at high concentrations for most cell types [48]),
- requirement of a set of strict quality controls to minimize the risk of contamination and to select appropriate FBS lots supporting growth of cells while retaining their regenerative and differentiation properties,
- interference of unidentified factors on the effect of hormones, growth factors, or other additives under investigation,
- limited availability,
- ethical issues [49].

Considered together, despite strict selection and testing for safety and growth-promoting capacity, the use of FBS represents a *major obstacle* for the wide implementation of hMSC-related therapies.

#### 3.3.2. Humanized Media

In order to alleviate the safety and regulatory concerns raised by the use of animal serum for generating hMSCs, autologous or allogeneic human blood-derived materials, including human serum, plasma, platelet derivatives (e.g., platelet lysate), and cord blood serum, are currently under investigation for their clinical utility as an alternative medium supplement.

Human autologous serum has been reported to support hMSC expansion [50–53]. It would be problematic, however, to acquire amounts sufficient to generate clinically relevant numbers of hMSCs. Moreover, the use of autologous serum may not be applicable for elderly patients as its capacity to support cell growth may decrease with their age. The performance of human allogeneic serum from adult donors is rather controversial because contradictory results have been reported [52, 54–58]. Allogeneic human serum from UCB [59, 60] and placenta [61] has also been proposed as potential alternatives to replace FBS because these primitive tissues are a rich source of growth factors [49].

Attempts have also been made by many investigators to examine the utility of human platelet lysate (hPL), which has been prepared by mechanical disruption or chemical lysis of the platelet membrane [49], for the cultivation of hMSCs. Most of the studies reported that the growth factor-enriched allogeneic hPLs have considerable growth-promoting properties for hMSCs while maintaining their differentiation potential and immunomodulatory properties [62–70]. However, some other studies reported data that showed a reduction of osteogenic or adipogenic differentiation potential when hMSCs were cultured in hPL-based media [67, 71]. Moreover, a recent report illustrated that, although cell proliferation was greatly enhanced, the use of hPL (supplemented into RMPI 1640 medium) altered the expression of some hMSC surface molecules and led to a decrease in their *in vitro* immunosuppressive capacity [72]. This study also showed that the production of prostaglandin E_2_, which has previously been demonstrated to play a major role in the suppression of immune cells [73], was lowered under the use of hPL compared to FBS.

Although considered relatively safer than FBS for human therapeutic applications, the use of human-sourced supplements is still a matter of substantial debate, prompting some concerns [74, 75]. There is a risk that allogeneic human growth supplements may be contaminated with human pathogens that might not be detected by routine screening of blood donors. Moreover, these crude blood derivatives are poorly defined and suffer from batch-to-batch variation, and thus their ability to maintain hMSC growth and therapeutic potentials could be widely variable. In particular, the variability can be a significant hindrance for implementing the clinical-scale production of hMSCs simply because it could make it difficult to obtain cells retaining desired qualities in a consistent and predictable manner, which is crucial for minimizing treatment failures.

#### 3.3.3. Defined Serum-Free Media

The concerns raised from the use of ill-defined serum or human-sourced supplements demonstrate the need for the development of defined serum-free media. While reducing the problems associated with such crude materials, defined serum-free media may further provide additional advantages as follows.

- Defined formulations designed to support the generation of a population enriched with a desired cell type (i.e., hMSCs) while preventing overgrowth of undesired cells in primary cultures will lead to the production of more homogeneous hMSCs (i.e., more precisely, a population of adherent cells containing a high content of colony-forming multipotent mesenchymal cells). It has been shown that medium formulations greatly affect the frequency and size of colonies in the primary and passaged hMSC culture, and a systematically optimized defined medium for hMSCs led to significant increased colony-formation compared to FBS-based cultures [76].
- The well-defined nature of the medium would facilitate enhancing cell bioprocessing protocols, which may be crucial for increasing clinical efficacy by producing cells with desired properties. For instance, based on the proposed mechanism that hMSCs exert therapeutic benefits via the secretion of certain soluble molecules, the medium formulations may need to be modified to enhance expression of specific genes to achieve an optimal cytokine profile [3].
- When *ex vivo* differentiated cells are desirable for therapeutic use, the transition of an expansion state to a differentiation phase under defined conditions could facilitate the production of such desired cell types in a more favorable and controllable environment. Davani et al. demonstrated that, in an effort to differentiate hMSCs (pancreas-derived) to insulin-expressing cells, the shift of a serum-based expansion condition to a serum-free differentiation condition led to considerable cell death [36]. In principle, it may be less harsh to cells to switch only key “growth-promoting factor(s)” to “differentiation-inducing factor(s)” while maintaining the base condition.

Attempts have been made to develop defined serum-free media for animal or human MSC growth; however, most of them have demonstrated only limited performance [77–80]. These media formulations were only shown to support cell expansion for single-passage cultures or at slow rates through multiple passages. Moreover, all of these studies used cells which had previously been exposed to serum during the initial isolation/expansion phases. Serum-derived contaminants are probably carried over with the cells when they are placed under serum-free conditions after exposure to serum, and thus, exposure to serum may ultimately limit their therapeutic use.

The ideal media should consist of chemically defined constituents that support the attachment and growth of hMSCs primary cultures as well as passaged cultures, while maintaining their therapeutic properties. Towards this objective, our group has recently carried out a study to identify key attachment and growth factors required for both primary and passaged cultures, and this study led to the development of a defined serum-free medium (PPRF-msc6) for hMSC isolation and expansion [76]. We demonstrated that PPRF-msc6 medium supported the generation of hMSCs from multiple BM samples in a rapid and consistent manner, maintaining their multipotency and hMSC-specific immunophenotype. Furthermore, compared to a classical serum-supplemented media (i.e., DMEM supplemented with prescreened FBS), hMSCs cultured in PPRF-msc6 exhibited numerous advantages from a production standpoint. Specifically, these hMSCs had a greater colony-forming capacity in primary as well as passaged cultures, negligible lag phase and explicit exponential growth, lower population doubling times (21–26 h versus 35–38 h; between passage levels 1 and 10), a greater number of population doublings (62 ± 4 versus 43 ± 2; over a two-month period), and a more homogeneous cell population, which was smaller in size [81]. Consequently, the sustained production of smaller hMSCs in a rapid manner requires less time and surface area to obtain clinically relevant numbers of hMSCs, while reducing risk of contamination and saving cost and labor. Moreover, from a therapeutic viewpoint, the size of cells to be transplanted into patients could be an important issue because it has been shown in animal-model studies that most of hMSCs grown in FBS-supplemented media were trapped in the lung [82]. Small hMSCs may offer a significant benefit in transplantation therapies because the small cells may travel through the lung and home to the site of injury or disease at high efficiencies [83]. Similar to the performance with BM cells, PPRF-msc6 also allowed for the isolation and extended expansion of hMSCs from other sources, including AT and pancreatic tissue samples, more rapidly and efficiently compared to control FBS-supplemented media [our unpublished data]. In view of the “defined” status, PPRF-msc6 contains some serum components, such as insulin, transferrin, serum albumin, and fetuin, which are often associated with traces of other serum constituents, and thus further investigations need to be conducted to refine this medium to a true chemically defined medium by replacing these components with synthetic alternatives. Recently, for instance, we have successfully replaced native insulin with a recombinant insulin without any decrease in the performance of PPRF-msc6 for hMSC culture. The replacement of native transferrin, albumin, and fetuin in this medium with recombinant alternatives or other supplements is currently underway. Nonetheless, PPRF-msc6 represents the most well-defined serum-free formulation to support both the isolation and expansion of hMSCs in the literature to date and a significant step forward for producing hMSC therapeutics. The protocol for PPRF-msc6 preparation has been described in detail [76] so that the disclosed formulation or its modifications can be further developed by any interested party.

Efforts have also been made to test or modify existing defined medium formulations designed for other stem cell types in order to cultivate hMSCs. Rajala et al. illustrated that a defined, xeno-free medium for human embryonic stem cells (hESCs) allowed, during a single-passage culture, the expansion of hMSCs previously isolated from AT samples in the presence of allogeneic human serum [84]. This study did not report whether this medium supported the growth of hMSCs in primary culture as well as through multiple passages. Also, Mimura et al. modified a defined hESC medium to promote the expansion of an immortalized genetically modified hMSC line [85]. The cells grown in their disclosed medium formulation demonstrated differentiation capacity towards osteogenic and adipogenic lineages while displaying a rather different gene expression profile compared to those cultured in FBS-based medium.

#### 3.3.4. Commercially Available Media for Expanding hMSCs

Several commercially available serum-free media have recently been introduced for the expansion of hMSCs [reviewed in [86]]. StemPro MSC SFM from Invitrogen represents the first commercial serum-free medium that allows the isolation and expansion of hMSCs from BM and has recently been cleared by the FDA as a medical device for clinical trials in the United States (http://www.invitrogen.com/). Agata et al. demonstrated that this medium supported hMSC growth more rapidly at early passages while reaching senescence earlier (at passage 5) with gradually reduced proliferation rate, compared to a control FBS medium [87]. In addition, although most of the hMSC-specific surface antigens were expressed on both cell populations expanded in the serum-free medium and an FBS-based reference medium, some molecules were expressed in different levels (i.e., CD105 and CD146). Moreover, it appears that both cell populations displayed different levels of stemness as well as different differentiation potential (i.e., cells expanded in serum-free medium exhibited lower ALP activity in noninduced state, but a greater response to osteogenic induction compared to serum-based controls). In summary, this commercial serum-free medium seems to generate hMSCs with different characteristics in comparison with those derived in classical FBS media.

Aiming towards the widespread implementation of hMSC-related therapy in later stage of clinical trials, serum-free, xeno-free media for hMSC culture have also been commercialized. However, the formulations of these commercial media are not disclosed, which may restrict their wide utility in hMSC research and clinical studies. The identification of specific medium components allowing for the serum-free isolation and expansion of hMSCs would contribute significantly to the research and therapeutic applications of these cells. Moreover, as medium formulations determine cell characteristics (i.e., growth pattern, gene expression, phenotype, and functional properties), it is important to evaluate carefully each of these commercial media for intended therapeutic applications, preferably in parallel to select the best choice for each specific target.

**Figure 1:**
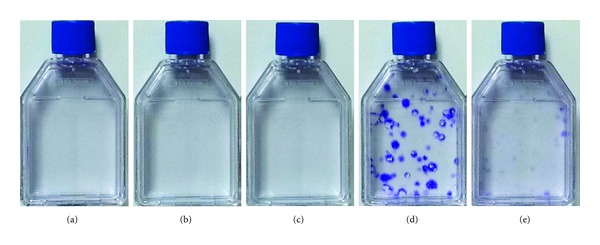
Cultivation of primary human BM MNCs using different media including three commercial media. Cells were inoculated at 150,000 BM MNCs/cm^2^ into fibronectin-coated T-25 flasks, each containing 8 mL of Mesencult-XF (a), StemPro MSC SFM Xeno-Free (b), MSCGM-CD (c), and PPRF-msc6 (d), and a classical FBS medium (10% FBS DMEM) (e). After 60 h, nonadherent cells in each medium were removed, and fresh medium was added to the adherent cells (100% medium change). The adherent cells were allowed to grow for additional 10 days with 50% medium change every other day, and then stained with crystal violet to visualize colonies generated.

We have recently initiated a study to compare these commercial media along with other existing media. The media under investigation include Mesencult-XF (STEMCELL Technologies), StemPro MSC SFM Xeno-Free (Invitrogen), MSCGM-CD (Lonza), PPRF-msc6, and DMEM +10% FBS (Lonza). Our initial data show that the performance of the commercial hMSC media on the attachment and growth of primary BM-hMSCs is questionable and thus should be open to discussion. Specifically, in a preliminary experiment, the commercial media were evaluated in parallel with PPRF-msc6 and 10% FBS DMEM by plating human BM MNCs into human fibronectin-coated T-25 flasks containing each of the media. Cells were inoculated at 150,000 cells/cm^2^, and nonadherent cells in each medium were removed after 60 hours with 100% medium change. Thereafter, the adherent cells were allowed to grow with 50% medium replacement every other day, and then stained on day 12. In this experiment, none of the commercial media demonstrated cell growth (Figures 1(a)–1(c)). In contrast, a significant number of well-developed colonies were found in the culture with PPRF-msc6 (Figure 1(d)). The serum-based control culture with 10% FBS DMEM also resulted in the formation of colonies although most of them were still premature. Lindroos et al. reported that StemPro MSC SFM Xeno-Free medium provided significantly higher proliferation rates of AT MSCs when compared with serum-containing media [88]. In their study, however, the authors tested the commercial serum-free medium using cells that were previously isolated and expanded in a serum-containing medium (10% human serum), and the ability of StemPro MSC SFM Xeno-Free medium to allow the growth of primary hMSCs was not addressed. Moreover, Hartmann et al. stated that they were not able to culture hMSCs derived from the UC tissue using StemPro MSC SFM Xeno-Free medium without serum [73]. In contrast, the authors demonstrated that Mesencult-XF medium supported the isolation and expansion of UC-hMSCs without the use of serum. In an attempt to culture hMSCs from BM using a xeno-free protocol (i.e., proprietary Mesencult-XF Attachment Substrate as well as medium), Miwa et al. also showed that the use of Mesencult-XF medium resulted in the growth of primary BM-hMSCs more rapidly than a serum-containing medium [89].

**Figure 2:**
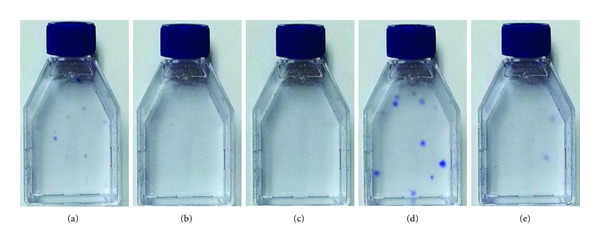
Cultivation of nonadherent cell fractions removed from the culture of primary BM MNCs in different media. Nonadherent cells and spent medium removed from each of the flasks, demonstrated in Figure 1, were replated into a new T-25 flask coated with gelatin containing 4 mL of the fresh medium—that is, Mesencult-XF (a), StemPro MSC SFM Xeno-Free (b), MSCGM-CD (c), PPRF-msc6 (d), and 10% FBS DMEM (e). After 60 h, nonadherent cells and medium in each flask were discarded, and fresh medium was added to the adherent cells (8 mL per flask). The adherent cells were allowed to grow for additional 8 days with 50% medium change every other day and then stained with crystal violet to visualize colonies generated.

The contradictory data between our work and the literature [73, 89] regarding Mesencult-XF medium may be due, at least in part, to the use of different substrate materials. Specifically, we used human fibronectin-coated flasks, while Miwa et al. used a proprietary substrate-coating product. In this regard, we plated the nonadherent cells, which were removed from the first medium change during the primary culture with each medium described earlier, into new flasks containing the same medium to allow them to attach to the new substrate and grow. For convenience, here we call these as “secondary” cultures as opposed to their original cultures described in the previous paragraph and Figure 1. The “secondary” flasks were coated with gelatin (bovine), which is widely used to facilitate the attachment of many types of anchorage-dependent cells to the substrate. In these secondary cultures, we observed that a number of colonies were formed in the culture with Mesencult-XF medium (Figure 2(a)). Together with the data obtained from the original culture (Figure 1(a)), this demonstrates the ability of Mesencult-XF medium to allow the growth of primary hMSCs, but implying that the performance of the medium could be improved by manipulating the substrate-coating materials. In contrast, StemPro MSC SFM Xeno-Free medium and MSCGM-CD medium did not allow for colony formation (Figures 2(b) and 2(c), resp.). These data suggest that factors required for the isolation of hMSCs from primary cultures seem to be missed in both media. Similar with the study by Hartmann et al. [73], we also observed that the addition of low serum (i.e., 2% prescreened FBS) into StemPro MSC SFM Xeno-Free medium (and MSCGM-CD medium) supported the growth of primary BM-hMSCs (data not shown). Based on these observations, therefore, we would argue that it should be desirable to further optimize all the commercial media described here for the serum-free, xeno-free isolation of hMSCs. Regarding PPRF-msc6, beyond the high number of large colonies generated in the original culture (Figure 1(d)), a number of colonies also appeared in the secondary cultures (Figure 2(d)), which were initiated with the nonadherent cells that had been normally discarded in our previous work [81]. In contrast, the FBS-based culture led to the appearance of only a few colonies in its secondary culture (Figure 2(e)), indicating that the majority of hMSCs obtainable from the use of 10% FBS DMEM attached to the surface of original culture in the presence of known and unknown attachment factors included in serum. Considered together, these data imply that the yield of hMSCs obtained from the primary culture of BM cells with PPRF-msc6 could further be increased by modifying the culture protocols, particularly by identifying optimal attachment factors and/or substrate-coating materials to enhance initial cell attachment efficiencies.

A commercial medium (mTeSR) with disclosed composition, which was originally developed for the expansion of hESCs, has also been tested for hMSC culture [90]. Although this defined medium together with human fibronectin-treated substrate allowed the expansion of BM-hMSCs previously isolated using FBS-based medium, it did not support cell growth in primary BM cultures. In addition, when hMSCs were plated into the mTeSR at a very low density for a CFU-F assay, the colonies derived were significantly smaller at lower frequency, compared to a control case with FBS-based medium. Moreover, mTeSR-derived cells demonstrated significantly decreased adipogenic potential. Therefore, further studies should be carried out with this medium to identify factors affecting multipotency as well as growth of hMSCs to make the disclosed formulation viable for research and clinical applications.

In summary, although numerous defined hMSC media are commercially available or have been introduced in the literature to support the growth of hMSCs, one must realize that the therapeutically relevant properties of culture-expanded hMSCs could be significantly affected by medium components. Considering the safety and efficacy required to produce hMSC therapeutics for intended clinical applications, it is crucial to compare different media (and their formulations if the recipe is disclosed) and probably further optimize the formulations in a systematic manner. In this regard, the disclosed medium formulations for hMSCs (e.g., those reported in [76, 80, 85, 90]) are best positioned to be further developed by the many investigators interested in therapeutic applications of hMSCs.

## 4. Development of Defined Serum-Free Media

Defined serum-free medium development or optimization for a specific cell type is a very complicated process because multiple variables that affect the maintenance, growth, and characteristics of cells are interrelated. Moreover, designing a new serum-free formulation for anchorage-dependent cells such as hMSCs tends to be more fastidious compared to those grown in suspension culture, as the interaction of cells with the substrate on which they attach and spread prior to growth needs to be understood. Medium development studies should involve rational approaches: (i) to select appropriate factors (e.g., basal medium formulations and growth/attachment proteins) and (ii) to screen them in a stepwise, systematic manner for their effect on cell properties and growth.

### 4.1. Cell Culture Requirements

An understanding of the requirements for successful cell culture is a prerequisite for designing a rational strategy towards the efficient development/optimization of a new serum-free medium. In addition, since anchorage-dependent hMSCs normally require serum, namely, some serum components yet unidentified but responsible for their attachment, spread, and growth, it is particularly crucial to understand the functions that serum serve in cell culture in order to identify such components. Hence, specific requirements of nutrients and nonnutrient elements for cell culture are briefly reviewed below with a special emphasis on the constituents and functions of serum.

#### 4.1.1. Nutrients

Nutrients refer to chemical substances that are taken into cells and utilized as substrates in energy metabolism or biosynthesis, as catalysts in those processes, or as structural components of cellular organelles. The nutrients are divided into organic nutrients, inorganic salts, and trace elements, and the organic nutrients are further subdivided into amino acids, carbohydrates, lipids, vitamins, and others [91]. Generally, the nutrients are considered as the “defined” portion of culture media. In addition to the “nutrient” roles, the nutrients also have regulatory functions. It has been demonstrated that these nutrients could represent the only requirements for *in vitro* growth of certain transformed cell lines [92]; however, more fastidious nontransformed normal cells typically require additional growth-promoting supplements for their growth in culture.

**Organic Nutrients**

Amino acids represent essential elements of media as building blocks for protein synthesis. In addition, certain amino acids have other key roles in multiplication of cells in culture, especially under serum-free conditions. In particular, glutamine appears to play major roles in many metabolic pathways, and thus an adequate extracellular concentration of glutamine is typically needed in cell culture media. It is common to add 2–4 mM of glutamine to hMSC media. It is important to note that glutamine is labile under cell culture conditions, and thus the amount of glutamine for hMSC culture should be determined considering both the requirement for cell growth and its breakdown during the culture. Moreover, nonessential amino acids have been added into a defined medium developed for hMSC growth [80].

A carbohydrate source is essential for the growth of cells in culture, since neither amino acids nor fats can readily be used either as the sole energy source or as substrates to build up a sufficient pool of intracellular carbohydrate intermediates. Glucose is the commonly provided source of energy for cells in culture, and together with amino acids it is included in most defined basal medium formulations. It is known that glucose at high concentrations has harmful effects on some cell types in culture. For this reason, low concentrations of glucose (~5 mM) are commonly used for hMSC culture. However, recent evidence reveals that high glucose concentrations (~25 mM) led to comparable or higher growth of animal and human MSCs [93–95].

It is known that certain lipids, such as cholesterol, free linoleic acid and its metabolites, free oleic acid, and phospholipids containing linoleic acid, stimulate growth of mammalian cells. Lipids are frequently not included in the defined portions of cell culture media. Large amounts of bound lipids are contained in serum; therefore, normally serum-containing media do not need the supplementation of additional lipids. In contrast, externally supplied lipids are generally required in serum-free culture [91]. Lipid supplements have been added to serum-free media for hMSCs [76, 80].

In general, mammals require 12 vitamins, including the 4 fat-soluble vitamins (A, D, E, and K) and the 8 members of the B complex (thiamine, riboflavin, niacin, pyridoxine, pantothenic acid, folacin, vitamin B_12_, and biotin). In addition, primates, guinea pigs and flying mammals require vitamin C (ascorbic acid). In contrast, normal diploid cells in culture exhibit requirements for the B vitamins, while some cells have shown growth responses to ascorbic acid. For this reason, most cell culture media include all the B vitamins but variably contain vitamin C [91]. Other vitamins are not included in media. The B vitamins function as cofactors for specific enzymes, and their deficiency can result in death of the animal. Ascorbic acid functions as an oxygen acceptor in several mixed-function oxidase systems. Rowe et al. reported that ascorbic acid had an effect on collagen synthesis by human diploid fibroblasts and played a role as a growth-promoting factor for many cell types [96]. The use of ascorbic acid for hMSC culture seems to be a matter of debate. Gronthos and Simmons demonstrated that ascorbic acid was a critical component under serum-free conditions for supporting the formation of CFU-F colonies in primary cultures of human BM cells [97]. We and others also demonstrated that ascorbic acid promoted hMSC growth [76, 98]. Moreover, it was observed that the lack of ascorbic acid in medium significantly reduced osteogenic potential of hMSCs [76]. On the other hand, ascorbic acid has typically been used as a supplement in some MSC differentiation media. Mimura et al. reported that ascorbic acid increased osteoblastic marker expression in hMSCs grown in a serum-free condition, and thus the authors removed this component from their defined medium formulation [85]. It should be noted that ascorbic acid is very labile under cell culture conditions [99]; therefore, care should be taken when this substance is used in cell culture.

**Inorganic Salts**

The salts that are included in most media are those of Na^+^, K^+^, Mg^2+^, Ca^2+^, Cl^−^, SO_4_^2−^, PO_4_^3−^, and HCO_3_^−^, and they play several functions. The salts primarily contribute to retaining the osmotic balance of the cells. The osmolality of most cell culture media is approximately 300 mOsm/kg, and this represents an optimal value for most cell lines. It has been shown that many cell lines tolerate variation of approximately 10% of this optimal value, and thus care should be taken when extra salts are added into a medium [45]. Divalent cations, particularly Ca^2+^, are required by some cell adhesion molecules, such as the cadherins. Ca^2+^ also acts as an intermediary in signal transduction, and the concentration of Ca^2+^ in the medium can have an influence on cell proliferation or differentiation. Na^+^, K^+^, and Cl^−^ regulate membrane potential, while SO_4_^2−^, PO_4_^3−^, and HCO_3_^−^ have roles as anions required by the matrix and nutritional precursors for macromolecules, as well as regulators of intracellular charge. HCO_3_^−^ also plays a role as a buffer and its concentration is determined by the concentration of CO_2_ in the gas phase [100].

**Trace Elements**

In addition to the inorganic salts, other inorganic elements, such as Mn^2+^, Cu^2+^, Zn^2+^, Mo^6+^, Va^5+^, Se^8+^, Fe^2+^, Ca^2+^, Mg^2+^, Si^4+^, Ni^2+^ are present in serum in trace amounts. These substances are referred to as trace elements and are included in most medium formulations. Although the role of these trace elements has been only partially elucidated, it has been demonstrated that many of these elements act as enzyme cofactors and are essential to the survival and growth of most cells [45, 101]. For instance, selenium is well recognized as an activator of glutathione peroxidase, a key enzyme essential for detoxifying cytotoxic oxygen radicals, and has been considered as an essential trace element for many cell types in culture [92, 102, 103]. It was also observed that selenium increased the proliferation of hMSCs from AT [104]. In contrast, numerous reports demonstrated that selenium suppressed cell proliferation in culture and induced cytotoxicity [105]. In our study, the addition of selenium into a serum-based medium reduced the colony-forming ability of hMSCs [76]. Considered together, the effect of selenium on hMSC expansion seems to depend on culture conditions or cell sources.

#### 4.1.2. Nonnutrient Factors

Beyond the nutrients, the growth of mammalian cells requires additional substances (provided from serum or other sources). These nonnutrient factors, including growth and attachment factors and hormones, generally function in regulatory roles on cellular differentiation as well as growth and proliferation. These regulatory factors are not included in most basal media and are frequently supplemented to serum-free media. Often, the requirements of nutrients for the growth of cells of interest could be satisfied by selecting appropriate defined basal media; therefore, identifying growth-stimulating regulatory factors has typically been the key subject in the development of serum-free media. Much effort has been made to investigate the effect of cytokines and growth factors on hMSC growth. Many reports showed that bFGF promotes the proliferation of hMSCs [76, 106–109]. TGF-*β*1 is also known to support hMSC proliferation in combination with other growth factors [85, 106]. It is important to note that TGF-*β*1 alone showed a growth-inhibitory effect on hMSCs, while demonstrating a significant degree of synergistic effect with bFGF [76]. The impact of PDGF on hMSCs is controversial. Several studies reported that PDGF enhanced the proliferation of MSCs from human and animal BM [77, 97, 106]. Moreover, PDGF-enriched plasma or platelet lysate has been shown to support the isolation and expansion of hMSCs. In contrast, our group observed that PDGF reduced considerably the colony-forming property of hMSCs [76]. It is presumed that the contradictory data are due, at least in part, to different modes of interactions with different factors present in different culture conditions. Other growth factors, such as EGF, Activin A, aFGF, and FGF4, have also been shown to promote hMSC proliferation (e.g., [76, 110, 111]); however, their effects could be masked in the presence of more potent factors (e.g., bFGF) [76].

Binding proteins such as albumin and transferrin are commonly added to serum-free media. Parker et al. reported that the absence of albumin in their serum-free formulation reduced hMSC growth [80]. Supplementation of key hormone components into serum-free media is also crucial. We observed no stimulatory effects of insulin and progesterone under FBS-based conditions in our study [76]. Nonetheless, these components showed growth-promoting effects for many cell types in serum-free condition [112]. Hydrocortisone has been shown to increase the proliferation of adherent human BM cells [113]. It has also been demonstrated that dexamethasone, a synthetic reagent of fluoridated hydrocortisone, is an essential component in serum-free medium for the growth of CFU-F colonies in primary cultures of human BM cells [97]. Interestingly, our experiments showed that the supplementation of hydrocortisone into an FBS-containing medium significantly inhibited cell proliferation and caused a change in cell morphology from spindle-shaped to cuboidal (data not shown); however, this hormone was found to be a key component in our serum-free media for hMSC growth, displaying a considerable combined effect with fetuin, particularly in primary culture [76]. This indicates that the effect of hydrocortisone is highly dependent upon culture conditions. Fetuin, a major plasma glycoprotein, has been used as a requirement for serum-free primary cultures of hMSCs and other cell types such as mouse fibroblast and epithelial cells [76, 114]. Heparin, a glycosaminoglycan that typically acts as an anticoagulant factor, has been shown to have proliferative or antiproliferative effects on various cell types [115, 116]. Addition of heparin into culture media led to a reduced growth of hMSCs from AT and BM [66, 76]. In contrast, Mimura et al. reported the growth-enhancing effect of heparin on a genetically modified hMSC line [85].

#### 4.1.3. Other Variables

For successful cell cultures, it is also important to control other key variables, particularly when serum-free media are used. Beyond the nutrients and nonnutrient factors, medium pH, osmolality, and partial pressure of dissolved gases in culture are important for cell growth. In addition, environmental conditions, including temperature and the nature of culture surface, have significant impacts. Finally, culture techniques, such as trypsinization and passaging protocol, are also important factors. Review of these variables on hMSCs in serum-free conditions is out of the scope of this paper.

### 4.2. Serum Components and Functions

Media for the culture of stem cells must provide all the essential requirements for cell survival and growth while maintaining their undifferentiated characteristics. These requirements (both defined and undefined) are normally provided by using serum in hMSC cultures. Serum is an extremely complex fluid, which is prepared by defibrination of plasma. It contains a broad spectrum of biological factors (Table 1) having physiologically balanced growth-promoting, growth-inhibiting, and/or differentiation-inducing activities [101, 112]. The important components of serum and their main functions are as follows [45, 48, 101, 112]:

- growth factors (e.g., PDGF, EGF, FGF, IGF-1, IGF-2) promoting cell proliferation: some of these factors may be cytostatic and induce differentiation;
- components of base membrane (e.g., fibronectin) and other adhesion factors (e.g., fetuin and hydrocortisone, particularly present in fetal serum) supporting cell attachment and spreading;
- trace elements (e.g., selenium), minerals, vitamins (e.g., ascorbic acid), lipids, and hormones (e.g., insulin, hydrocortisone): many of these are bound to carrier proteins, stimulating cell growth, and are involved in many other biological activities;
- other nutrients (e.g., amino acids, nucleosides): some of these are present in solution and the others are bound to proteins. These nutrient components are largely included in basal media, but serum also provides necessary nutrients that may not be present in basal media or may not be present in sufficient amounts to promote growth;
- binding proteins (e.g., albumin, transferrin) carrying minerals, vitamins, lipids, hormones, and other nutrients: these proteins play a role to stabilize and modulate the activity of the components which they bind;
- buffer (e.g., albumin and others) modulating pH: the role of serum for increasing the buffering capacity is particularly important where the seeding density is low (e.g., cell cloning experiments);
- protease inhibitors (e.g., *α*2-macroglobulin) neutralizing proteases: these antiproteases protect cells from damage caused by their exposure to proteases such as trypsin used in the passaging procedure or proteases released by the cells during culture. They may also promote cell attachment;
- protection factors (e.g., albumin and others) contributing to viscosity and thus protecting circulating cells from mechanical damage (e.g., shear stress induced by pipetting or agitation in suspension culture): these elements are considered less important in monolayer culture, but they may be important in protecting trypsinized cells from the pipetting;
- antitoxins: these factors bind and neutralize toxins.

When serum is omitted from culture media, it is important to find substitutes (i.e., alternative medium supplements together with appropriate culture protocols) that can replace the serum functions essentially required for cell survival and proliferation. In most cases, the requirements are multiple, and thus it is necessary to investigate the effects of both nutritional factors and regulatory factors. Typically, native proteins purified from serum (e.g., albumin, insulin, transferrin, fetuin) and synthetic substances (e.g., recombinant growth factors) are added into serum-free formulations. The requirements of these and other supplements vary greatly, depending on the cell type being studied and the basal medium selected. Moreover, it is very important to employ appropriate culture protocols (e.g., culture surface, trypsinization) to properly examine the effects of the supplements.

### 4.3. Proposed Approaches for Defined Serum-Free Medium Development

Typically, to select basal media, growth factors and other medium supplements in a logical way (i.e., understanding serum factors and their functions, and finding related information from the literature) and then to perform trial and error experiments may be the only method to identify the best candidates. The most well-known fundamental strategies for the development of a new serum-free medium were proposed in the 1960s and 1970s by separate groups. Together with these classical methods, some practical approaches are summarized below.

#### 4.3.1. Ham's Approach

Ham developed a method for substantially reducing (or eliminating in certain cases) the amount of serum in a medium. This approach is based on the careful manipulation of media components and culture conditions (i.e., by modifying the defined constituents of an existing medium formulation and a culture protocol previously developed for a related cell type without adding growth factors) in order to provide cells with an optimal nutrient balance [92]. In other words, the adjustment of concentrations of the nutrients to optimum values and the manipulation of culture protocols enables significant reduction of the concentration of serum proteins, although it is unknown what functions of serum are exactly replaced. It has been demonstrated that variables whose modification has contributed to reducing the requirement for serum proteins include (i) the nature of the culture surface, (ii) the type of trypsinization procedure, (iii) buffering, (iv) pH, (v) osmolarity, (vi) the availability of all nutrients, and (vii) quantitative adjustment of their concentrations. The basic concept of Ham's approach to reduce serum in the medium is (i) to reduce the amount of serum to a level that restricts growth and then (ii) to look for changes in the medium or culture conditions that will improve growth. During their work, Ham and colleagues found it important to reexamine, at lower serum concentrations, those factors that had no effect on growth at higher amounts of serum.

**Procedure of Ham's Approach**

In the attempt to replace the functions of serum with the quantitative adjustment of the nutrient concentrations and culture conditions, Ham and colleagues used the following approach.

1. The amount of serum is reduced to a level that yields suboptimal growth in a medium. The medium is selected, based on its performance for the growth of a related cell type.
2. To identify those components whose quantitative adjustment is the most limiting to growth with the low concentration of serum, the effect of increasing and decreasing the concentration of each individual component of the medium by 5 or 10 times is tested.
3. The component that has the greatest effect in this preliminary survey is then tested over a wide range of concentrations. In this step, a typical growth-response curve is generated and divided into three parts as follows:
  - The first is a direct growth response, in which growth improves as the nutrient concentration is increased until a saturation value is reached.
  - The second is a plateau where further increases in the nutrient concentration have no effect on growth.
  - The third is a toxic response in which increasing the nutrient concentration has a detrimental effect on growth.

**Figure 3:**
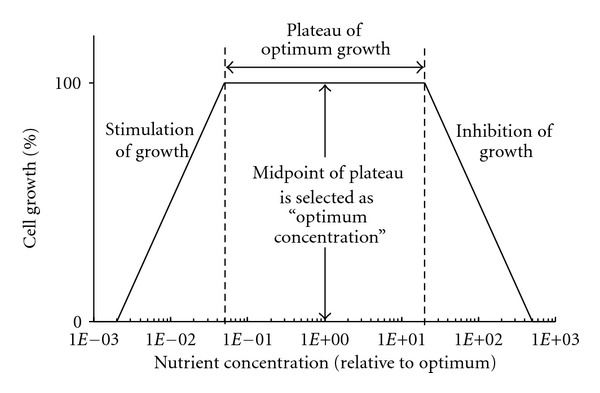
Idealized growth response curve versus nutrient concentration illustrating the procedure for determining the “optimum” concentration of a nutrient. The range of concentrations that support optimum growth (referred to as a “plateau”) is determined, and its midpoint on the plot is selected as the concentration to be used in future media (Adapted from [92]).

Figure 3 shows a schematic growth-response curve typical for most components, although the growth stimulation to certain factors is biphasic in rare cases [92]. At the upper end of the curve in Figure 3, serum proteins have the ability to protect cells from the inhibitory effect of excess amounts of nutrients. At the lower end of the curve, a large amount of serum proteins permit cells to grow at concentrations of essential nutrients that would be inadequate with lesser amounts of serum protein. Each time that such a titration is performed, the midpoint of the plateau region on the growth response curve is selected as the optimum concentration for use in future media. This process is useful to keep the selected nutrient concentration as far separated as possible both from nutritional inadequacy and from toxicity (i.e., the lower end and upper end, respectively, of the growth-response curve in Figure 3).

(4) Once the quantitative adjustment improves growth, the concentration of serum is reduced until it again becomes limiting.
(5) At the readjusted lower concentration of serum, the next most critical component whose concentration needs to be adjusted is determined repeating the steps 2 and 3.
(6) These steps above are repeated until serum is reduced to the minimum level or is completely eliminated in certain cases of transformed cells.

Based on the results obtained using this approach, Ham and colleagues classified the growth-promoting functions of serum into two operational categories, “replaceable” and “nonreplaceable”. The replaceable category consists of those functions of serum that could be replaced by making changes in the defined portion of the medium or in the culture conditions. In contrast, the nonreplaceable functions are those that could not be replaced using this approach. Ham's group reported that serum was completely eliminated by using their method for the growth of some cell lines including certain normal cells; however, it was later found that these cell lines underwent subtle transformations, which enabled their growth in the absence of serum proteins [117].

Using this approach, Ham's group was able to formulate a variety of basal media, such as Ham's F12 and MCDB series, providing optimal nutrient balances to certain cell types. These medium formulations supported the clonal growth of many specific cell lines in the presence of a minimized amount of serum. However, this approach is extremely labor intensive and time consuming. Further, as the “nonreplaceable functions” of serum could not be replaced for the growth of normal cells using this approach, externally supplied growth-promoting substances, in the form of dialyzed serum, purified fractions from serum, or synthetic materials, should still be provided to the cells to obtain satisfactory growth even with the optimized media and culture conditions.

#### 4.3.2. Sato's Approach

In contrast to Ham's approach, which is analytical in nature, Sato and colleagues developed a synthetic method for the replacement of serum in culture media. This approach was based on attempts (i) to understand what roles serum play for the maintenance and growth of cells in culture and then (ii) to supplement an existing basal medium formulation with a combination of key hormones and growth factors mimicking the growth-stimulatory function of whole serum while restricting the manipulation of the basal media components [48]. For example, one of the main functions of serum is to provide a mixture of hormones, which is stimulatory for cell growth. In order to identify the active additives, an array of factors is tested under suboptimal conditions—for example, at lowered serum concentrations, as described below.

**Procedure of Sato's Approach**

1. The growth promoting capacity of serum is first lowered to a level that yields suboptimal growth by reducing the concentration of serum, in order to see a stimulation of growth when various factors under investigation are added to the medium.
2. Upon understanding what functions serum serve for cells in culture and the specific requirements of nutrients and nonnutrient growth factors, a large array of factors is selected and tested under the suboptimal conditions.
3. As active factors are identified, the serum concentration is further lowered and the search continued.

Through their extensive work on many cell types, Sato's group identified various key essential supplements, hormones, binding proteins, lipids, trace elements, and attachment factors, required for addition to basal medium. In particular, they demonstrated that insulin, transferrin, and selenium were essential for the growth of most cells while hydrocortisone and EGF were additionally needed for certain cell types. Using their approach, Sato and colleagues were able, for a number of different types of cells, to replace serum with hormones and growth factors while leaving the basal medium essentially unchanged. However, Sato's approach is still labor intensive and time consuming.

#### 4.3.3. Top-Down and Bottom-Up Approaches

Considered as more practical approaches, top-down and bottom-up approaches can be used effectively for the development of a new serum-free medium formulation for the growth of a cell population of interest [45].

**Top-Down Approach**

This approach involves employing an existing medium formulation for a similar cell type, and identifying stimulatory components in the presence of serum for the growth of the target cells. This process proceeds as the concentration of serum is gradually reduced. This concept evolved from the premise that a cell type, which belongs to a group of cells with similar characteristics, often requires the same combination of growth factors for growth. When this approach is used, care should be taken to identify the existence of any cytotoxic or growth-inhibitory components in the medium for the cells being studied.

**Bottom-Up Approach**

This approach involves first selecting an appropriate basal medium (e.g., a medium used for the growth of a related cell type) and then screening various selected exogenous factors for their growth-stimulatory effects. Since only the active components required for the growth of cells of interest are added into the medium, the final formulation will represent an efficient and easily amendable medium. However, this approach is likely to be labor-intensive and time-consuming. Further, since the screening of factors being examined is performed in the absence of serum, the critical functions of serum required to see their effects should be carefully considered and satisfied by alternative means (e.g., well-controlled physiochemical parameters, treatment of culture surface, trypsinization and passage protocols), because normally the serum-free basal medium does not provide such functions.

**Figure 4:**
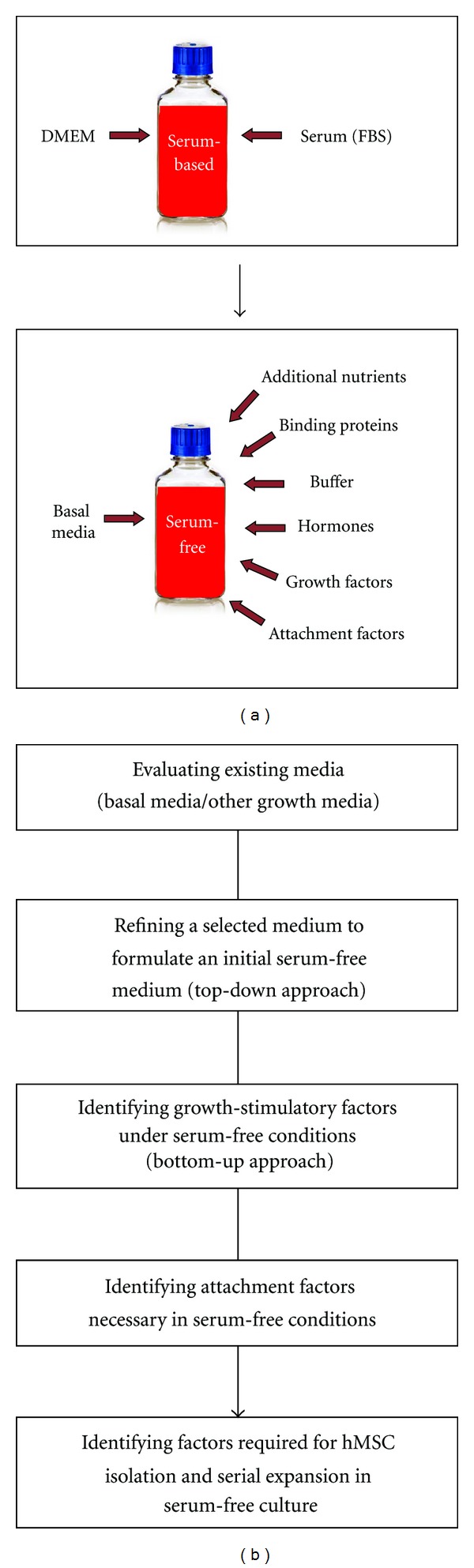
An overview demonstrating a process for the development of a defined serum-free medium for hMSCs. (a) To replace ill-defined serum with defined supplements, a variety of medium constituents, including basal media, additional nutrients (e.g., lipids and vitamins), binding proteins, physiochemical reagents (e.g., buffer), hormones, growth factors, and attachment factors were selected. (b) A sequential strategy was designed for screening effectively the selected basal media and medium additives to develop a defined serum-free condition that supports the isolation and expansion of hMSCs [76].

We would like to point out that there is no universal guideline for screening selected medium additives towards the development of a new medium, and thus it is important for investigators to understand the advantages and disadvantages of all the approaches previously proposed and then to exploit beneficial features of each approach for designing their own strategies in a rational, effective manner. As an example, in our study for hMSC serum-free medium development [76], we selected various medium ingredients (basal media, extra nutrients, binding proteins, buffering agents, hormones, vitamins, and growth and attachment factors) based on the understanding of cell culture requirement including the role of serum constituents. And then the selected factors were examined in a sequential manner, employing some useful suggestions of each approach described earlier, in order to determine chronologically their impact on proliferation, attachment, and isolation of hMSCs (Figure 4). In addition, an effective serum-free medium development can be achieved by considering other important issues as discussed below.

### 4.4. Considerations for the Development of a New Serum-Free Medium

#### 4.4.1. Selection of Materials and Testing Methods

**Selection of Basal Media**

hMSCs are typically cultured in Dulbecco's Modified of Eagle's Medium (DMEM) or Minimum Essential Medium Alpha (*α*MEM) with the supplementation of FBS. However, whether these basal media are appropriate for serum-free culture should be critically considered for the cell type being investigated, because each basal medium has been developed or optimized for a specific application. For example, Ham's F12, which was developed for the clonal growth of Chinese Hamster Ovary cells in low serum, contains a wide range of ingredients at low concentrations, while DMEM, which was optimized at higher cell densities for viral propagation, contains fewer constituents but at high concentrations [45]. For this reason, a 1 : 1 mixture of DMEM and Ham's F12 has been used for the culture of many cell types with serum or as a basis for serum-free media, since this combination provides a reasonable compromise between high concentrations and a wide range of ingredients. The DMEM/F-12 mixture has been employed as a basal medium for the development of serum-free media for hMSCs [76, 80].

**Selection of Factors to Be Tested**

Recent trends towards the development of serum-free media for the growth of a cell type often exclusively focus on the effect of “regulatory growth factors” such as peptide growth factors and hormones. When an established serum-free medium exists for those cells or a closely related cell type, this approach represents a reasonable method, because this type of study is more likely to be characterized as a medium optimization or modification process, rather than a “significant” development. When such a medium is not available, the medium development process will be more extensive and complicated. In this case, together with the wide-ranging investigation on growth-promoting growth factors and other medium supplements, considerations should also be placed on other properties of cells (e.g., attachment, in particular in primary culture). In this regard, an understanding of serum functions and related constituents is important to select candidates to be examined.

**Culture Protocol for Screening Factors**

In the development of serum-free media for anchorage-dependent cells, as described earlier, conventional approaches for screening growth factors include the use of serum with gradual reduction of its content in media to (a) provide unidentified adherent proteins for facilitating cell attachment to the substrate and (b) support sufficient growth levels to observe meaningfully the impact of the additives [48, 92]. However, this procedure is very labor intensive and time consuming. As a more practical approach, it has been suggested to plate the cells initially in serum-containing medium, remove the medium after the cells attach, and screen the factors in serum-free medium [48]. It was further recommended that this protocol would work best in the presence of insulin and transferrin because these two components are required for most cell types and their presence is necessary for the appearance of a stimulatory effect of other factors [48]. This screening protocol has also been effectively employed for a serum-free medium development for hMSCs [76].

**Necessity of Reexamination of Factors**

Through their extensive work on medium development, both Ham and Sato demonstrated that the composition of a medium formulation could mask effects of certain factors under screening that could reveal their impact at different medium compositions or their concentrations [48, 92]. For example, it was shown that some factors, such as transferrin, were not stimulatory until the serum concentration was substantially reduced [48]. This is because some serum components at high concentrations covered the effect of transferrin, which became unmasked at the decrease of their concentrations to certain levels. Conversely, effects of certain factors under screening could also be unrevealed in the absence of some medium components. Therefore, both Ham and Sato proposed that the examination of selected factors and the adjustment of their concentrations to optimum values be done in a stepwise manner at progressively defined compositions and concentrations of the medium formulation and that certain factors having no effects at a condition be reevaluated under a revised screening condition.

**Synergistic Effects**

Growth factors often act synergistically or additively with each other or with other hormones. In this regard, statistical approaches have been widely used to investigate specific interactions between growth factors under screening. This method will be powerful when a good screening medium, which includes requirements for cell maintenance and at least “minimal” growth as well as attachment, is available to examine effectively the individual and synergistic effects of the selected growth factors.

**Contamination with Other Trace Elements**

It is known that purified serum proteins, such as albumin, insulin, transferrin, and fetuin, often carry other trace components, which may affect cell growth. Therefore, it is desirable to use highly purified substances or completely defined synthetic materials, if available, to determine conclusively the effect of such proteins.

#### 4.4.2. Cell Culture Aspects in Serum-Free Conditions

**Cell Handling**

Cells grown in serum-free conditions are more delicate than those grown in the presence of serum and thus should be handled very gently to minimize cell damage during the harvesting and passaging procedures.

**Buffer System of Media**

Since the buffering function of serum to modulate pH is omitted in serum-free cultures, it may be beneficial to further supplement the medium with a chemical buffer such as HEPES, in addition to the bicarbonate-CO_2_ system, in order to improve the buffering capacity of the medium. The HEPES concentration could be increased above 15 mM without toxicity for some cell lines, but it may be necessary to adjust the osmolality of the medium accordingly [48].

**Lack of Detoxifying Substances**

As serum proteins that could bind and neutralize toxic contaminants are not present in serum-free conditions, its protective, detoxifying activity is also omitted. Thus, care should be taken in selecting water, reagents, as well as culture techniques. The *level of purity of water and reagents and the degree of cleanliness of all apparatus must be very high* [45, 48, 112]. In general, basal media are recommended to be kept at 4°C no longer than 2 weeks because their constituents are more likely to be decomposed in the absence of those detoxifying serum components. Growth factors and other medium supplements, in particular labile components such as transferrin, hormones, and ascorbic acid, should be reconstituted, stored and used strictly according to the manufacturer's instruction.

**Lack of Protease Inhibitors**

The addition of serum to cells exposed to trypsin during trypsinization neutralizes any residual proteolytic activity. Protease inhibitors such as aprotinin and soybean trypsin inhibitor could be used to replace this function. However, the level of their antiproteolytic action and their potential impact on cell growth should be examined by testing these materials in a dose-dependent manner in comparison with a control case using serum. In addition, as an alternative trypsinization protocol, the use of native trypsin could be replaced by a less detrimental protease (e.g., recombinant trypsin) for cell harvesting. Moreover, recombinant trypsin has effectively been used for hMSC culture in serum-free conditions [84, 87–90]. When recombinant trypsin is used to detach cells from the substrate, the use of FBS or other trypsin inhibitors may not be necessary for serum-free culture. In contrast, Hudson et al. used serum albumin (1%) in PBS to wash cells after trypsinization with recombinant trypsin [90].

## 5. Conclusions

Clinical efficacy for the use of hMSCs has been variable and probably still insufficient for widespread implementation of hMSC therapies. Enhancing culture protocols may be a critical issue to meet efficacy endpoints in upcoming clinical studies. Well-formulated chemically defined serum-free media for hMSC isolation and expansion would greatly contribute to the achievement of this goal. Towards this objective, significant progress has been made to generate various medium formulations for hMSC culture in the absence of ill-defined FBS and human-sourced supplements. These defined media should be critically evaluated through *in vitro* and *in vivo* analyses and most likely further refined for optimal performance. In this regard, fully disclosed formulations should represent important platforms for enhancing the therapeutic potential of hMSCs. We also emphasize that the identification of key elements towards the development and optimization of serum-free media should follow rational, systematic approaches in order to maximize the possibility of finding their true effects on hMSCs. All the issues reviewed herein should thus be considered seriously when medium development and optimization studies are carried out.

## Figures and Tables

**Table 1:** Constituents of serum [Adapted from [112]].

	Range of		Range of
Constituent	concentration	Constituent	concentration^a^
Proteins and polypeptides	40–80 mg/mL	Polyamines:	0.1–1.0 *μ*M
Albumin	20–50 mg/mL	Putrescine, Spermidine	
Fetuin^b^	10–20 mg/mL		
Fibronectin	1.0–10 *μ*g/mL	Urea	170–300 *μ*g/mL
Globulins	1.0–15 mg/mL		
Protease inhibitors:	0.5–2.5 mg/mL	Inorganics	0.14–0.16 M
*α*l-antitrypsin,		Calcium	4.0–7.0 mM
*α*2*-*macroglobulin		Chlorides	100 *μ*M
Transferrin	2.0–4.0 mg/mL	Iron	10–50 *μ*M
		Potassium	5.0–15 mM
Growth factors:		Phosphate	2.0–5.0 mM
EGF, PDGF, IGF1 and 2,	1.0–100 ng/mL	Selenium	0.01 *μ*M
FGF, IL-1, IL-6		Sodium	135–155 mM
		Zinc	0.1–1.0 *μ*M
Amino acids	0.01–1.0 *μ*M		
		Hormones	0.1–200 nM
Lipids	2.0–10 mg/mL	Hydrocortisone	10–200 nM
Cholesterol	10 *μ*M	Insulin	1.0–100 ng/mL
Fatty acids	0.1–1.0 *μ*M	Triiodothyronine	20 nM
Linoleic acid	0.01–0.1 *μ*M	Thyroxine	100 nM
Phospholipids	0.7–3.0 mg/mL		
		Vitamins	0.01–10 *μ*g/mL
Carbohydrates	1.0-2.0 mg/mL	Vitamin A	10–100 ng/mL
Glucose	0.6–1.2 mg/mL	Folate	5.0–20 ng/mL
Hexosamine	0.6–1.2 mg/mL		
Lactic acid	0.5–2.0 mg/mL		
Pyruvic acid	2.0–10 *μ*g/mL		

^a^The range of concentrations is approximate and is intended to convey only the order of magnitude.

^b^In fetal serum only.
